# The links between agriculture, *Anopheles* mosquitoes, and malaria risk in children younger than 5 years in the Democratic Republic of the Congo: a population-based, cross-sectional, spatial study

**DOI:** 10.1016/S2542-5196(18)30009-3

**Published:** 2018-02

**Authors:** Mark M Janko, Seth R Irish, Brian J Reich, Marc Peterson, Stephanie M Doctor, Melchior Kashamuka Mwandagalirwa, Joris L Likwela, Antoinette K Tshefu, Steven R Meshnick, Michael E Emch

**Affiliations:** a Duke Global Health Institute, Duke University, Durham, NC, USA; b Department of Geography, Chapel Hill, NC, USA; c Department of Biostatistics, Chapel Hill, NC, USA; d Carolina Population Center, Chapel Hill, NC, USA; e Department of Epidemiology, Chapel Hill, NC, USA; f University of North Carolina, Chapel Hill, NC, USA; g President's Malaria Initiative and Entomology Branch, Division of Parasitic Diseases and Malaria, Center for Global Health, Centers for Disease Control and Prevention, Atlanta, GA, USA; h Department of Statistics, North Carolina State University, Raleigh, NC, USA; i Hopital General Provincial de Reference, Kinshasa School of Public Health, Kinshasa, Democratic Republic of the Congo; j Programme National de Lutte contre le Paludisme, Kinshasa, Democratic Republic of the Congo; k Department of Community Health, Ecole de Santé Publique, Faculté de Médecine, University of Kinshasa, Kinshasa, Democratic Republic of the Congo

## Abstract

**Background:**

The relationship between agriculture, *Anopheles* mosquitoes, and malaria in Africa is not fully understood, but it is important for malaria control as countries consider expanding agricultural projects to address population growth and food demand. Therefore, we aimed to assess the effect of agriculture on *Anopheles* biting behaviour and malaria risk in children in rural areas of the Democratic Republic of the Congo (DR Congo).

**Methods:**

We did a population-based, cross-sectional, spatial study of rural children (<5 years) in the DR Congo. We used information about the presence of malaria parasites in each child, as determined by PCR analysis of dried-blood spots from the 2013–14 DR Congo Demographic and Health Survey (DHS). We also used data from the DHS, a longitudinal entomological study, and available land cover and climate data to evaluate the relationships between agriculture, *Anopheles* biting behaviour, and malaria prevalence. Satellite imagery was used to measure the percentage of agricultural land cover around DHS villages and *Anopheles* sites. *Anopheles* biting behaviour was assessed by Human Landing Catch. We used probit regression to assess the relationship between agriculture and the probability of malaria infection, as well as the relationship between agriculture and the probability that a mosquito was caught biting indoors.

**Findings:**

Between Aug 13, 2013, and Feb 13, 2014, a total of 9790 dried-blood spots were obtained from the DHS, of which 4612 participants were included in this study. Falciparum malaria infection prevalence in rural children was 38·7% (95% uncertainty interval [UI] 37·3–40·0). Increasing exposure to agriculture was associated with increasing malaria risk with a high posterior probability (estimate 0·07, 95% UI −0·04 to 0·17; posterior probability [estimate >0]=0·89), with the probability of malaria infection increased between 0·2% (95% UI −0·1 to 3·4) and 2·6% (–1·5 to 6·6) given a 15% increase in agricultural cover, depending on other risk factors. The models predicted that large increases in agricultural cover (from 0% to 75%) increase the probability of infection by as much as 13·1% (95% UI −7·3 to 28·9). Increased risk might be due to *Anopheles gambiae* sensu lato, whose probability of biting indoors increased between 11·3% (95% UI −15·3 to 25·6) and 19·7% (–12·1 to 35·9) with a 15% increase in agriculture.

**Interpretation:**

Malaria control programmes must consider the possibility of increased risk due to expanding agriculture. Governments considering initiating large-scale agricultural projects should therefore also consider accompanying additional malaria control measures.

**Funding:**

National Institutes of Health, National Science Foundation, Bill & Melinda Gates Foundation, President's Malaria Initiative, and Royster Society of Fellows at the University of North Carolina at Chapel Hill.

## Introduction

Understanding the ecology of malaria and its vectors is an essential component of successful malaria control.^1^ In sub-Saharan Africa, agriculture is an important aspect of this ecology.^2^, ^3^ Agriculture is of concern because more than half of the global population growth from now to 2050 is expected to occur in Africa,^4^ and UN projections suggest the population could double, from 1·2 billion in 2015 to 2·5 billion in 2050, with much of this growth occurring in rural areas.^5^, ^6^ Such growth places considerable demand on Africa's food supply, and governments are considering large-scale agricultural projects to meet this increased need.^6^, ^7^ However, agricultural projects might reverse reductions in malaria transmission that have been achieved over the past decade, because expanding agriculture might produce habitat characteristics favoured by *Anopheles gambiae* sensu lato mosquitoes, sub-Saharan Africa's most efficient malaria vectors. Specifically, agricultural uses might increase the availability of the pools of water with little or no surrounding vegetation that are the preferred breeding sites for *A gambiae* sensu lato mosquitoes.^8^, ^9^, ^10^

Few studies collect data on both vector populations and malaria prevalence. Entomological studies tend to focus on the relationship between the environment and transmission-related indicators. Findings from these studies suggest that agricultural development is associated with changes in mosquito indoor resting density, human biting rates, sporozoite rates, entomological inoculation rates, larval abundance, reproduction rates, gonotrophic cycles, and vector capacity.^11^, ^12^, ^13^, ^14^, ^15^, ^16^, ^17^ Such changes, however, do not necessarily increase malaria risk. Increased larval density, for example, is associated with longer larval development times,^15^, ^18^ and different agricultural practices and crop types have had varying effects on malaria risk.^7^, ^19^, ^20^ Importantly, individual studies tend to be done in a small number of sites, are not representative of a country's broader population, and are not generalisable across its ecological zones. They also frequently have a paucity of data on other key factors governing transmission, such as bednet use, limiting inferences on risk.^21^ Therefore, given the diversity of vectors and human ecosystems, additional work in this area is needed.

**Research in context**

**Evidence before this study**

The relationship between agriculture, mosquito populations, and malaria risk in human beings is complex. We searched PubMed and Google Scholar with the terms “agriculture”, “anopheles”, “land use”, “land use change”, and “environment” in combination with “malaria”. We applied no language or publication date restrictions. Agriculture has been consistently associated with several malaria-related transmission parameters, such as indoor resting density, human biting rates, entomological inoculation rates, larval abundance, gonotrophic cycles, and vector capacity. By contrast, no consistent effect of agriculture on malaria risk has been found. However, these studies tend to be done in a small number of sites, and are not representative of the broader population or across different ecological zones. Additionally, we found these studies also often had a paucity of data on important factors that govern transmission.

**Added value of this study**

To our knowledge, this is the first study attempting to understand the agriculture–malaria relationship across multiple ecological zones using population-based human survey data, contemporaneous mosquito vector surveillance, and satellite imaging.

**Implications of all the available evidence**

Our results show that increasing agricultural land cover increases the probability of infection with *Plasmodium falciparum* malaria with high posterior probability across ecologically diverse settings. This increase in infection probability might be mediated by changes in the biting behaviour of *Anopheles gambiae* sensu lato mosquitoes. Interestingly, bednets impregnated with permethrin were not protective whereas bednets impregnated with deltamethrin were protective. Malaria control programmes and policy makers must consider potential increases in malaria risk due to expanding agriculture.

In this study, we examine the relationship between agriculture, the mosquito population, and malaria risk in children living in the Democratic Republic of the Congo (DR Congo)—a large and ecologically diverse country containing 47% of Africa's potential agricultural land and accounting for 10% of global malaria deaths in 2015^6^, ^22^—and we consider possible mechanisms through which increases in agriculture might lead to a hypothesised increase in malaria risk.

## Methods

### Study design and population

We did a population-based, cross-sectional, spatial study of children from the DR Congo. The study population comprised children younger than 5 years sampled as part of the 2013–14 DR Congo Demographic and Health Survey (DHS), which is a population-based cluster household survey. Because the DHS did not provide survey information about children aged 5 years or older, these children were excluded. Additionally, we included only children living in rural areas (as defined by the DHS, which uses each country's definition) because agriculture in the DR Congo is predominantly rural. The sampling methods for the DHS are described elsewhere.^23^ We used information about the presence of malaria parasites in each child, as determined by PCR analysis of dried-blood spots according to a previously published protocol.^24^, ^25^, ^26^ Dried-blood spots were obtained from the DHS for malaria DNA extraction, of which some samples were randomly selected for use for other projects and were therefore excluded from this study. We also excluded samples that were negative for human β tubulin.

Parental consent for children's participation in the 2013–14 DHS was obtained by the DHS Programme. The 2013–14 DR Congo DHS was reviewed and approved by the institutional review board at ICF International—a global consulting firm and the contractor responsible for implementing the DHS survey—and the University of Kinshasa (Kinshasa, DR Congo). This study was approved by the institutional review board at the University of North Carolina (Chapel Hill, NC, USA).

### Exposure to agriculture

We derived measures of agricultural cover using the Moderate Resolution Imaging Spectroradiometer Land Cover Type data product (MCD12Q1), which provides yearly estimates of land cover at 500-m resolutions. In that dataset, two different classification schemes measured agricultural land cover: the International Geosphere-Biosphere Programme measure, which includes two agricultural land cover classes, and the University of Maryland measure, which includes one agricultural land cover class. We estimated the proportion of agricultural land cover within 10 km of each DHS cluster using each of the classification schemes, and then averaged the two estimates to lessen the effect any extreme measures in one classification might have on inference. We chose a 10-km radius because it corresponds to the maximum flight distance of a female, human blood-fed *A gambiae* mosquito, representing the maximum extent in which human and mosquito populations interact.^27^

### Population, behavioural, and environmental confounders

We derived population, behavioural, and environmental confounders from the DHS and satellite remote sensing sources. We extracted data from the DHS that consisted of age, sex, individual and community bednet use, altitude, and household construction materials, which represent both socioeconomic status and paths or barriers to mosquito entry. Individual bednet use was measured as use of a net treated with deltamethrin or alphacypermethrin, permethrin, or other kind of net (ie, nets for which we could not identify the insecticide). We consider net use in this manner because of high numbers of observed insecticide resistance to permethrin and remaining efficacy of deltamethrin and alphacypermethrin.^28^ Similarly, we calculate community bednet coverage according to the proportion of other respondents in the community sleeping under a deltamethrin-treated or alphacypermethrin-treated net, because these nets can still kill mosquitoes on contact. Household wall construction was coded as natural, rudimentary, finished, or other material according to the DHS. Roof construction was dichotomised as either finished (eg, metal or tin) or not, because of the small sample sizes in the rudimentary and other categories.

Precipitation and temperature were derived from multiple satellite platforms. We calculated the average temperature in °C the month the survey was done using the University of East Anglia's Climate Research Unit TS3.23 data product, together with data from the National Centers for Environmental Prediction and the National Oceanic and Atmospheric Administration. Precipitation was measured as the total rainfall in cm the month before the survey with use of Tropical Rainfall Monitoring Mission and the University of East Anglia's Climate Research Unit data. We calculated and averaged these measures within a 10-km radius of each survey cluster. Several studies have investigated different lag periods for precipitation and their effects on malaria transmission, with important lags identified ranging from 1 month to 5 months.^29^, ^30^, ^31^, ^32^ Therefore, we chose a lag of 1 month to be consistent with other studies of malaria in the DR Congo.^33^

### Entomological monitoring

We consider the effect of agriculture on the vector population using entomological surveillance of *A gambiae* sensu lato, *Anopheles paludis, Anopheles moucheti* sensu lato, *Anopheles funestus* sensu lato, and *Anopheles nili*. In 2013, the Africa Indoor Residual Spraying Project did two rounds of mosquito surveillance in August and November across four sites chosen to represent equatorial, tropical, and mountainous ecological regions of the DR Congo. In 2014, three more sites were added, yielding seven total sites for 2014 surveillance, which occurred in February, April, and July. One of these sites was in an urban setting (Kinshasa, DR Congo), and was excluded since interest is in rural transmission. Mosquito collection occurred both indoors and outdoors with use of human landing catch (HLC). HLC was done in eight households in each site. Households were chosen with use of convenience sampling, with an effort to select houses that were not immediately adjacent to one another. HLC was done in two households each night for four nights by two mosquito collectors between 1800 h and 0600 h, for a total of eight person-nights per site. One collector did HLC indoors and the other outdoors. The two collectors switched places hourly to prevent mosquito attraction bias. Identification of mosquito species was done morphologically.

Our outcome of interest is whether or not a mosquito was caught indoors. We assume mosquitoes caught indoors were intending to bite; therefore, we treated them as indoor-biting mosquitoes. We used the same strategy to measure agricultural cover, temperature, and precipitation around mosquito surveillance sites as the one we used for the DHS survey.

### Statistical analysis

We used probit regression to assess the relationship between agriculture and the probability of malaria infection. We fitted three models to assess the relationship between agriculture and malaria risk using DHS data. The three models addressed the survey sampling design, the unobserved vector population, and variability in crop types, with unobserved vector population and variability in crop types representing confounding sources. The first model incorporates an independently varying random intercept to account for the correlation induced by the survey's cluster sampling design. Such a model assumes unmeasured confounders exhibit no spatial structure. Given that the vector population is dependent on environmental conditions, which are spatially structured, we extended this model and incorporated spatial correlation in the intercept, thereby allowing for inference of unmeasured confounding variables across the DR Congo. Notably, both specifications assume no unmeasured confounding variables in the agriculture–malaria relationship. However, there might be variability in the effect due to different crop types, and different vectors might respond to agriculture in different ways. Therefore, we introduce a spatially varying coefficient process for the agriculture–malaria relationship.^34^

To generate a sense of how large-scale agricultural expansion might affect children younger than 5 years in the DR Congo, we used the best-fitting model output to plot the hypothetical probability of malaria infection for each child as a function of agriculture, with coverage ranging from 0% to 75% (ie, the minimum and maximum observed values in the data). We stratified each unique child according to their risk on the basis of other covariates from the model. To aid visualisation, we further stratified these children according to whether their risk fell into the lowest 25%, middle 50%, or highest 25% quantiles (ie, IQRs).

We also used probit regression models to assess the relationship between agriculture and the probability that a mosquito was caught biting indoors. Three separate models were fitted for indoor biting behaviour among *A gambiae* sensu lato, *A paludis*, and *A funestus* sensu lato mosquitoes. Insufficient numbers of *A moucheti* sensu lato and *A nili* mosquitoes prevented modelling. All three models included a random intercept that varied independently across surveillance sites, and controlled for temperature, precipitation, and month of surveillance.

We fitted all models in a Bayesian setting. Continuous covariates (age, agriculture, temperature, precipitation, and community bednet coverage) were first mean-centred and scaled, such that regression coefficients represent effects per SD increase in these variables. We assigned standard normal prior distributions to regression coefficients, and spatial structure was modelled using a Gaussian process with exponential covariance, consistent with other spatial models of malaria transmission.^35^, ^36^ We initially withheld a third of the data, and assessed performance of models on malaria risk using Brier scores, area under the receiver operating characteristic curve, and deviance information criterion. Final inferences were based on the best fitting model. The appendix provides a full discussion about the model specifications.

All data management and model fitting were done using R (version 3.3.1).

### Role of the funding source

The funders of the study had no role in study design, data collection, data analysis, data interpretation, or writing of the report. The corresponding author had full access to all the data in the study and had final responsibility for the decision to submit for publication.

## Results

**Figure 1:**
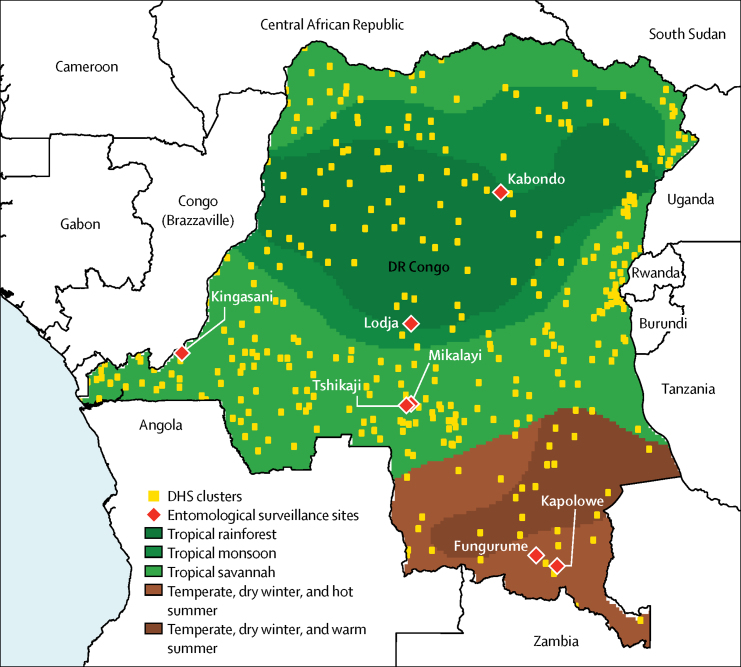
Study flow diagram QC=quality control. DHS=Demographic and Health Survey.

**Figure 2:**
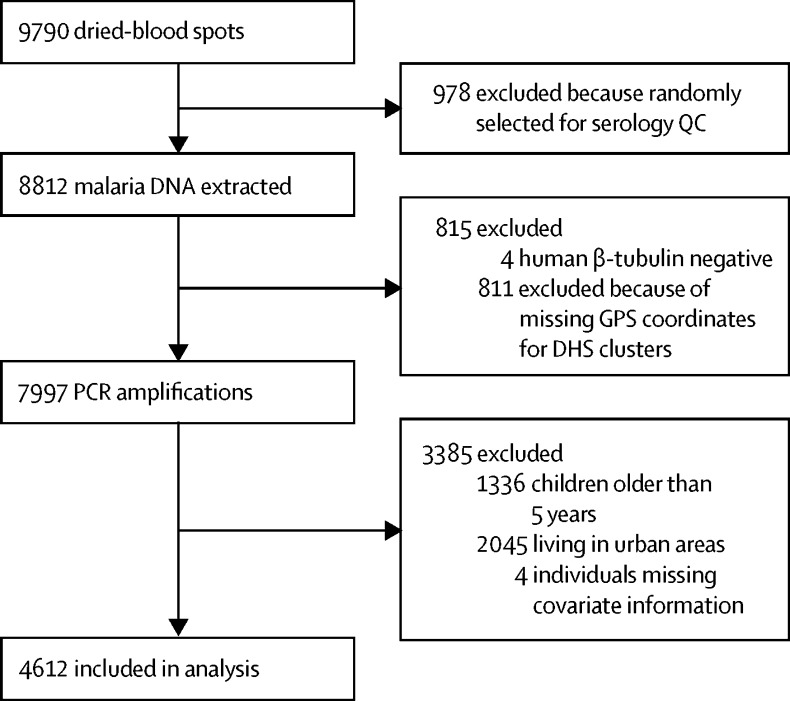
DHS survey sites, entomological surveillance sites, and the DR Congo ecoclimate regions The Kingasani site was excluded from the analysis because it was in the DR Congo capital of Kinshasa, a major urban centre with more than 10 million inhabitants. Ecoclimate regions are based on the Köppen–Geiger Climate Classification.^37^ DHS=Demographic and Health Survey. DR Congo=Democratic Republic of the Congo.

Between Aug 13, 2013, and Feb 13, 2014, a total of 9790 dried-blood spots were obtained from the DHS, of which 8812 were included for malaria DNA extraction. Four of these samples were negative for human β tubulin and were excluded, and spatial information was unavailable for 44 DHS clusters, reducing the sample size to 7997. Accounting for the other exclusion criteria, only 4612 participants in 331 survey clusters were included in this study (figure 1). Figure 2 shows the DHS and entomological surveillance sites identified and used in this study.

Prevalence of infection with *Plasmodium falciparum* malaria in rural children was 38·7% (95% uncertainty interval [UI] 37·3–40·0). Table 1 presents descriptive statistics on malaria infection prevalence according to the agricultural exposure and potential confounders, together with their expected relationships on malaria risk. Malaria-infected and malaria-uninfected children were exposed to almost the same agricultural land cover, temperature, and precipitation. Malaria prevalence was 7·5% higher in children living at altitudes less than 1000 m than in those living at 1000 m or more. Children sleeping under a bednet the previous night tended to sleep under nets treated with deltamethrin or alphacypermethrin. Among the 2829 malaria-negative children, 1367 (48·3%) slept under a net treated with deltamethrin or alphacypermethrin compared with 665 (37·3%) of 1783 children who tested positive for malaria. Whereas in children sleeping under permethrin-treated nets, a relatively increased proportion of malaria-positive children (165 [9·3%]) were reported compared with malaria-negative children (191 [6·8%]). Furthermore, malaria-negative children tended to live in communities with higher numbers of community bednet protection (47·1%) than were malaria-positive children (39·2%). Malaria-positive children also tended to live in poorer quality housing, with 157 (36·2%) sleeping in homes with finished wall construction compared with 277 (63·8%) of malaria-negative children.

**Table 1:** Descriptive statistics for variables included in probit models

		**Malaria-positive children (n=1783)**	**Malaria-negative children (n=2829)**	**Expected relationship to malaria**
**Individual-level variables**				
Mean age (years)		2·9 (SD 1·26)	2·6 (SD 1·30)	Increase risk
Sex				
	Girls	860 (48·2%)	1417 (50·1%)	Increase risk
	Boys	923 (51·8%)	1412 (49·9%)	Decrease risk
Bednet use				
	Deltamethrin or alphacypermethrin	665 (37·3%)	1367 (48·3%)	Decrease risk
	Permethrin	165 (9·3%)	191 (6·8%)	Unknown risk
	Other	37 (2·1%)	32 (1·1%)	Unknown risk
	No net	916 (51·4%)	1239 (43·8%)	Increase risk
Household wall material				
	Natural	245 (13·7%)	617 (21·8%)	Increase risk
	Rudimentary	1370 (76·8%)	1873 (66·2%)	Increase risk
	Finished	157 (8·8%)	277 (9·8%)	Decrease risk
	Other	11 (0·6%)	62 (2·2%)	Unknown risk
Household roof material				
	Natural	1574 (88·3%)	2408 (85·1%)	Increase risk
	Rudimentary	11 (0·6%)	16 (0·6%)	Increase risk
	Finished	196 (11·0%)	397 (14·0%)	Decrease risk
	Other	2 (0·1%)	8 (0·3%)	Unknown risk
**Community-level variables**				
Mean community bednet use* (%)		39·2% (SD 26·8)	47·1% (SD 28·7)	Decrease risk
Altitude				
	Children living <1000 m	1562 (87·6%)	2265 (80·1%)	Increase risk
	Children living ≥1000 m	221 (12·4%)	564 (19·9%)	Decrease risk
Mean precipitation (cm)		16·2 (SD 5·5)	16·5 (SD 4·8)	Increase risk
Mean temperature (°C)		24·7 (SD 1·4)	24·4 (SD 2·1)	Increase risk
Mean agricultural land cover (%)		11·1% (SD 15·5)	11·2% (SD 14·8)	Increase risk

Data are n (%) or mean (SD).

* Defined as the proportion of other community members sleeping under a deltamethrin-treated or alphacypermethrin-treated bednet.

Among the three models fitted to the DHS data, the model with an independently varying intercept yielded the best fit to the data. The appendix presents the fit statistics. Table 2 presents results from the best-fitting model, parameter estimates, 95% UIs, and the posterior probability that the exposure increases malaria risk. Increasing exposure to agriculture was associated with increased malaria risk with a high posterior probability (estimate 0·07, 95% UI −0·04 to 0·17; posterior probability [estimate >0]=0·89), with a 15% increase in agricultural cover associated with increased probabilities of malaria infection ranging from 0·2% (95% UI −0·1 to 3·4) to 2·6% (–1·5 to 6·6), depending on other risk factors such as bednets treated with deltamethrin or alphacypermethrin, age, housing quality, and altitude.

**Table 2:** Results for final probit regression model on agriculture and malaria risk

		**Estimate**	**2·5% UI**	**97·5% UI**	**Posterior probability (estimate >0)**
**Individual-level variables**					
Intercept		−0·34	−0·51	−0·16	0·00
Age (years)		0·18	0·14	0·23	1·00
Girls		0·03	−0·06	0·11	0·73
Bednet use (reference is no net)					
	Deltamethrin or alphacypermethrin	−0·15	−0·25	−0·05	0·00
	Permethrin	0·02	−0·17	0·21	0·58
	Other	0·19	−0·18	0·56	0·84
**Community-level variables**					
Household wall material (reference is natural)					
	Rudimentary	0·11	−0·04	0·27	0·92
	Finished	0·05	−0·18	0·29	0·66
	Other	−0·26	−0·77	0·26	0·17
Finished household roof material		−0·12	−0·29	0·06	0·09
Community bednet use (*Z* score)		−0·21	−0·31	−0·12	0·00
Altitude (>1000 m)		−0·30	−0·70	0·11	0·07
Precipitation (*Z* score)		−0·07	−0·19	0·04	0·11
Temperature (*Z* score)		0·17	0·03	0·32	0·99
Agricultural land cover (*Z* score)		0·07	−0·04	0·17	0·89

Posterior probability (estimate >0) values near or at 0 indicate that the effect is protective, whereas values at or near 1 indicate that the covariate is a risk factor. Values near 0·5 indicate no effect. UI=uncertainty interval.

**Figure 3:**
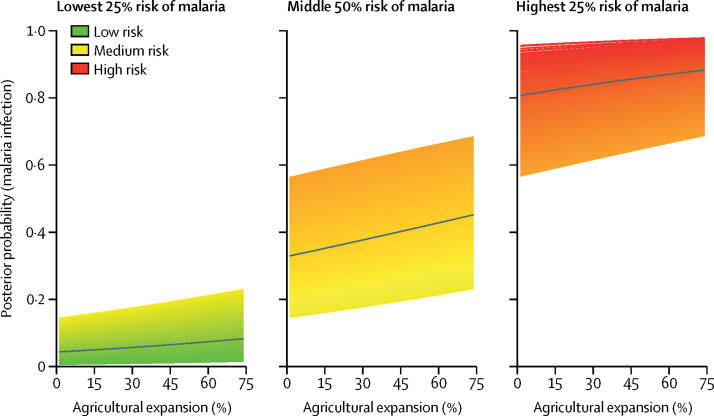
Hypothetical changes in malaria risk due to large-scale agricultural expansion in children younger than 5 years in the DR Congo The black line represents the mean trend lines within each quantile. DR Congo=Democratic Republic of the Congo.

Figure 3 plots the hypothetical change in malaria risk as a result of a large-scale agricultural expansion from 0% to 75% coverage. As shown, children at the extremes—ie, those at very low or very high risk for malaria based on other risk factors—exhibit a small increase in risk due to large-scale agricultural expansion. For those whose risk is not at either extreme, however, substantial increases in agriculture are accompanied by sizeable increases in malaria risk, as high as 13·1% (95% UI −7·3 to 28·9), indicating increases in malaria risk due to potential large-scale agricultural development might be offset through simultaneous investments in housing quality, bednets, and other interventions.

**Figure 4:**
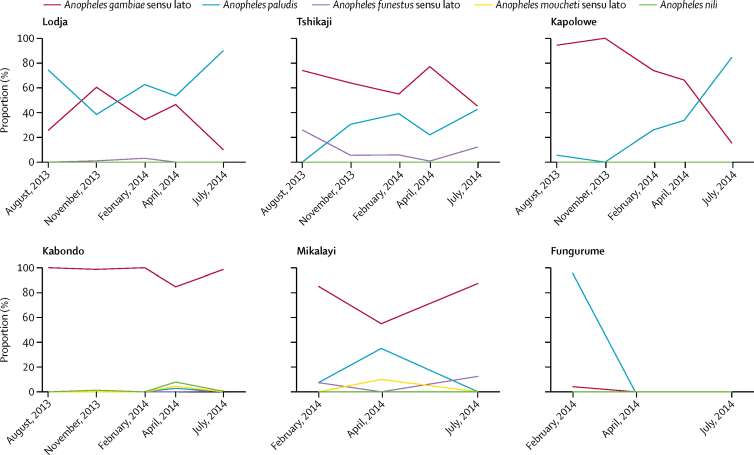
Relative abundance of Anopheles mosquitoes by human landing catch by site

*A gambiae* sensu lato and *A paludis* were the dominant mosquitoes collected across all sites and periods (table 3). *A funestus* sensu lato, *A moucheti* sensu lato, and *A nili* were relatively rare across all sites and times. Furthermore, relative abundance between *A gambiae* sensu lato and *A paludis* varied in some sites. In the Kapolowe site, relative abundance of *A gambiae* sensu lato declined over the course of surveillance, during which time abundance of *A paludis* increased, although relative abundance appears unrelated to season (figure 4)**.**

**Table 3:** Proportion of each *Anopheles* species by collection period of human landing catch

	**Collection period**					**Total caught**
	August, 2013	November, 2013	February, 2014	April, 2014	July, 2014	
*Anopheles gambiae* sensu lato	327 (56·4%)	630 (83·2%)	1320 (59·6%)	639 (63·0%)	761 (35·9%)	3677
*Anopheles paludis*	240 (41·4%)	120 (15·9%)	862 (38·9%)	350 (34·5%)	1328 (62·8%)	2900
*Anopheles funestus* sensu lato	13 (2·2%)	5 (0·6%)	32 (1·4%)	1 (0·1%)	25 (1·2%)	76
*Anopheles moucheti* sensu lato	0	0	0	10 (1·0%)	0	10
*Anopheles nili*	0	2 (0·3%)	0	14 (1·4%)	2 (0·1%)	18
Total caught	580	757	2214	1014	2116	6681

Data are n (%), unless otherwise stated.

Agricultural coverage across all six sites ranged from 3·7% to 25·3% (mean 15, SD 7), while total precipitation ranged from 0 to 265 cm (mean 79, SD 948). Average temperatures ranged from 19 to 26°C (mean 24, SD 2). Results from the probit models assessing the probability that an *Anopheles* mosquito was caught biting indoors varied across species. Among *A gambiae* sensu lato mosquitoes, increasing exposure to agriculture was associated with increased probability of biting indoors with a high posterior probability (estimate 0·50 95% UI −0·55 to 1·52; posterior probability [estimate >0]=0·84; table 4), controlling for available confounders. Given a 15% increase in agricultural cover, for example, this estimate was associated with increased probabilities of *A gambiae* sensu lato mosquitoes being caught biting indoors ranging from 11·3% (95% UI −15·3 to 25·6) to 19·7% (–12·1 to 35·9), depending on factors such as season (month of surveillance), temperature, and precipitation. Conversely, there was only a slight indoor biting response to agriculture in *A paludis* mosquitoes (posterior point estimate 0·15 [95% UI −0·93 to 1·17]; posterior point [estimate >0]=0·62). In *A funestus* sensu lato, increasing agriculture is associated with decreased probability of being caught biting indoors with a high posterior probability (estimate −0·72, 95% UI −2·33 to 0·82; posterior probability [estimate <0]=0·82). However, this mosquito species was not present in high abundance in any site. Table 4 presents the full model results, with parameter estimates, 95% UIs, and the probability that each variable increases indoor biting.

**Table 4:** Results of probit regression models assessing the effect of agriculture on indoor biting behavior in the DR Congo

		***Anopheles gambiae* sensu lato**				***Anopheles paludis***				***Anopheles funestus* sensu lato**			
		Estimate	2·5% UI	97·5% UI	Posterior probability (estimate >0)	Estimate	2·5% UI	97·5% UI	Posterior probability (estimate >0)	Estimate	2·5% UI	97·5% UI	Posterior probability (estimate >0)
Intercept		−0·78	−1·06	−0·50	0·00	−1·29	−1·59	−0·99	0·00	−0·51	−1·40	0·34	0·13
Agriculture		0·50	−0·55	1·52	0·84	0·15	−0·93	1·17	0·62	−0·72	−2·33	0·82	0·18
Precipitation		−0·15	−0·22	−0·09	0·00	0·31	0·07	0·57	0·95	0·15	−0·80	1·11	0·21
Temperature		−0·44	−0·59	−0·29	0·00	0·20	−0·04	0·07	0·99	−0·26	−0·90	0·39	0·61
Month													
	August, 2013 (ref)	··	··	··	··	··	··	··	··	··	··	··	··
	November, 2013	0·74	0·46	1·02	1·00	−0·57	−1·10	−0·04	0·02	−0·48	−2·12	1·15	0·29
	February, 2014	1·01	0·74	1·28	1·00	0·49	0·20	0·77	1·00	0·70	−0·49	1·92	0·87
	April, 2014	1·19	0·95	1·44	1·00	1·42	1·00	1·83	1·00	−0·50	−2·25	1·17	0·29
	July, 2014	0·58	0·39	0·78	1·00	0·69	0·41	0·96	1·00	0·32	−0·97	1·61	0·68

Posterior probability (estimate >0) values near 1 indicate high probability of increased indoor biting. Values near 0 indicate high probability of decreased indoor biting, whereas values near 0·5 correspond to little or no effect. UI=uncertainty interval.

## Discussion

Our data suggest that increasing agriculture is associated with increased malaria risk. This relationship does not meaningfully vary over space because of confounding variables from the unobserved vector population or crop types. Our model suggests that exposure to large-scale agricultural expansion will have a minimal effect on those with low risk of infection or where malaria infection rates are saturated. However, it could have profound effects on those not at either of these extremes. Such an effect is of concern in the DR Congo, which has the largest proportion of potentially available cropland in sub-Saharan Africa as well as one of the world's highest malaria burdens.

Results from our entomological analyses suggest that increases in agriculture are associated with increased probability of indoor biting among *A gambiae* sensu lato mosquitoes, but not among *A paludis*, and is associated with decreased probability of indoor biting in *A funestus* sensu lato. Given the high abundance of *A gambiae* sensu lato, these results suggest that the agriculture–malaria relationship might be mediated through effects on indoor biting among *A gambiae* sensu lato; and despite *A funestus* sensu lato showing a decreased probability of indoor biting with increasing agriculture, it only accounted for 1% of the mosquitoes collected. That said, given the preference for these mosquitoes to occupy larger, semi-permanent or permanent bodies of water, we hypothesise that the expansion of agriculture in these areas might lead to a decrease in the *A funestus* population, where it might be replaced by *A gambiae*. Important seasonal patterns also existed among vectors, with the relative abundances of *A paludis* and *A gambiae* sensu lato varying in some sites, while indoor biting behaviour among both species also varied, peaking in April, 2014.

Considerable work will be needed to fully understand the relationship between agriculture and malaria risk in sub-Saharan Africa. Studies on human adults are limited, with one study in the DR Congo finding no effect between agriculture and malaria risk.^33^ Additionally, the relationship between agriculture, temperature, and precipitation needs additional examination. In this study, we treat them as confounders, but they might also mediate risk, and their roles are complex.^32^, ^38^, ^39^, ^40^, ^41^, ^42^, ^43^, ^44^ That complexity, however, is not fully captured here. The irrigation scheme supplying water to agricultural land surrounding each survey cluster is a further unknown variable, and this is an important factor that deserves attention in future studies. How different crops affect malaria risk also deserves further consideration, although our modelling efforts indicated that the effect of any agriculture did not vary spatially, which in turn suggests that the crops present in the DR Congo increase risk. Finally, it is difficult to representatively sample the vector population over such a large land area, although our population was sampled in different ecological zones.

Work is also needed to understand the role of *A paludis*, which has received little attention in the malaria literature. Recent work to identify Africa's predominant malaria vectors predicted that *A gambiae* sensu lato was the dominant vector in the DR Congo, consistent with our data.^8^ However, work from the 1990s suggested that *A paludis* might be an important vector in the DR Congo, and given its observed presence and the country's high malaria burden, its role should not be discounted.^45^, ^46^

In conclusion, this work provides the first evidence that increased exposure to agriculture increases malaria risk in children younger than 5 years across rural and ecologically diverse settings, and might be due to increased indoor biting rates among *A gambiae* sensu lato mosquitoes. This finding is an area of growing concern for public health as transmission declines,^1^ and as governments consider initiating large-scale agricultural projects to respond to population growth. Such projects should be accompanied by additional malaria control measures; for example, environmental management, which has proven effective in reducing transmission in many different contexts.^7^, ^10^, ^47^
