# Supplementary material for: The links between agriculture, Anopheles mosquitoes, and malaria risk in children younger than 5 years in the Democratic Republic of the Congo: a population-based, cross-sectional, spatial study
Source: Lancet Planet Health. 2018 Feb;2(2):e74–82. doi: 10.1016/S2542-5196(18)30009-3 (PMC5809714; doi:10.1016/S2542-5196(18)30009-3)
Supplement: Supplementary appendix [file mmc1.pdf]

THE LANCET
Planetary Health

Supplementary appendix

This appendix formed part of the original submission and has been peer reviewed. We post it as supplied by the authors.

Supplement to: Janko M M, Irish S R, Reich B J, et al. The links between agriculture, Anopheles mosquitoes, and malaria risk in children younger than 5 years in the Democratic Republic of the Congo: a population-based, cross-sectional, spatial study. *Lancet Planet Health* 2018; **2:** e74–82.

Supplemental Material

STROBE Statement—Checklist of items that should be included in reports of *cross-sectional studies*

	Item No	Recommendation
**Title and abstract**	1	(*a*) Indicate the study's design with a commonly used term in the title or the abstract<br><br>**We state that this is a cross-sectional study in the title.**
		(*b*) Provide in the abstract an informative and balanced summary of what was done and what was found<br><br>**We describe the study populations, outcome measures, exposure measures, statistical methods, and results of our analysis in the abstract.**
**Introduction**		
Background/rationale	2	Explain the scientific background and rationale for the investigation being reported<br><br>**Scientific Background: We provide a comprehensive summary of previous work on the relationship between agriculture and malaria, noting that much of this work focuses either on the mosquito population or the human population, but not both. We further note that these studies tend to be conducted in a small number of settings, limiting generalizability. Given the diversity of human ecosystems and of vectors, we argue that more work on the role of agriculture in malaria transmission is needed.**<br><br>**Rationale: We write in the introduction that Africa's population is expected to double in the coming decades, with such population growth necessitating further development of the region's agricultural sector. Such development, however, may slow or reverse recent progress in reducing malaria transmission since agricultural development produces habitat characteristics favoured by *Anopheles gambiae* s.l. mosquitoes. Thus, we argue that better understanding this ecology is a critical component of future malaria control.**
Objectives	3	State specific objectives, including any prespecified hypotheses<br><br>**We state that our objective is to "examine the relationship between agriculture, the mosquito population, and malaria risk using data from a population-based cross-sectional survey of children under 5 years of age living in the Democratic Republic of Congo…and contemporaneous entomological monitoring data collected over time across DRC's ecological zones."**

		**We state that our hypothesis is that increasing exposure to agriculture is associated with increased malaria risk, and seek to understand how changes in vector behaviour may be a mechanism underlying this hypothesized increase.**
**Methods**		
Study design	4	Present key elements of study design early in the paper<br><br>**We state both in the title and in the introduction that this is a cross-sectional study. We further describe the study populations (children under 5 years of age and *Anopheles* mosquitoes) in detail, including sample sizes and selection criteria in the methods section.**
Setting	5	Describe the setting, locations, and relevant dates, including periods of recruitment, exposure, follow-up, and data collection<br><br>**We describe in detail the country setting, and include a map (Figure 2) showing where both the survey was conducted and where entomological surveillance occurred.**
Participants	6	(*a*) Give the eligibility criteria, and the sources and methods of selection of participants<br><br>**We describe the eligibility criteria in detail in the methods section. Briefly, they are children under 5 years of age living in rural DRC and mosquitoes sampled across six rural sites in different ecological zones in the DRC.**
Variables	7	Clearly define all outcomes, exposures, predictors, potential confounders, and effect modifiers. Give diagnostic criteria, if applicable<br><br>**We define outcomes and exposures in the description of each study population, and dedicate a separate section to confounding variables and how they were measured.**

Data sources/ measurement	8*	For each variable of interest, give sources of data and details of methods of assessment (measurement). Describe comparability of assessment methods if there is more than one group<br><br>**We describe the source of each variable (outcome, exposure, or confounder). We further state that the confounders common to both study populations were measured identically.**
Bias	9	Describe any efforts to address potential sources of bias<br><br>**We address sources of bias in previous work, how our work addresses such biases, and further discuss possible bias in our work. We address potential sources of bias in our work methodologically through the use of hierarchical Bayesian spatial models (including spatially-varying coefficient processes), and further by noting other limitations/sources of bias that in the discussion.**
Study size	10	Explain how the study size was arrived at<br><br>**We provide a description of the selection criteria and provide a study flow diagram as Figure 1 in our study.**
Quantitative variables	11	Explain how quantitative variables were handled in the analyses. If applicable, describe which groupings were chosen and why<br><br>**We provide a detailed description of how quantitative variables were handled (e.g. centering and scaling).**
Statistical methods	12	(*a*) Describe all statistical methods, including those used to control for confounding<br><br>**We provide a detailed description of our statistical methods, including a supplementary appendix that provides the Markov Chain Monte Carlo (MCMC) algorithm for each model considered.**
		(*b*) Describe any methods used to examine subgroups and interactions

		**We did not consider subgroups or interactions**
		(*c*) Explain how missing data were addressed<br><br>**We state that this is a complete case analysis, as there were only 4 study subjects out of 4,616 with any missing data. There was no missing data in the mosquito population.**
		(*d*) If applicable, describe analytical methods taking account of sampling strategy<br><br>**We provide a detailed description of the methodological approach, and how the model specifications address the both the sampling strategy and different sources of potential unmeasured confounding.**
		(*e*) Describe any sensitivity analyses<br><br>**We implement 3 different hierarchical Bayesian models to investigate possible sensitivity of our main findings due to unmeasured confounding. We describe the rationale for these model specifications in the *Statistical Analyses* section of the manuscript. We further describe how we assess model fit and provide fit statistics in the accompanying appendix.**
**Results**		
Participants	13*	(a) Report numbers of individuals at each stage of study—eg numbers potentially eligible, examined for eligibility, confirmed eligible, included in the study, completing follow-up, and analysed<br><br>**This information is included in the introduction**
		(b) Give reasons for non-participation at each stage<br><br>**The mothers for all eligible participants assented to their children being included in the study.**

		(c) Consider use of a flow diagram<br><br>**We include a flow diagram in the introduction**
Descriptive data	14*	(a) Give characteristics of study participants (eg demographic, clinical, social) and information on exposures and potential confounders<br><br>**We include this information in Table 1, where we also include the expected relationship between the variable of interest on malaria risk.**
		(b) Indicate number of participants with missing data for each variable of interest<br><br>**Only 4 individuals out of 4,616 had missing data, and we thus**
Outcome data	15*	Report numbers of outcome events or summary measures<br><br>**We begin the results section by summarizing malaria prevalence of under-5 children living in rural communities of the DRC.**
Main results	16	(*a*) Give unadjusted estimates and, if applicable, confounder-adjusted estimates and their precision (eg, 95% confidence interval). Make clear which confounders were adjusted for and why they were included<br><br>**We do not include a discussion of unadjusted estimates owing to space limitations. Additionally, our literature review indicated that confounding is an important limitation of studies on the agriculture-malaria relationship, and we therefore focus on addressing this confounding both by including confounders that are otherwise unavailable in other studies, and by exploring possible remaining unmeasured confounding through the use of Bayesian spatial models.**
		(*b*) Report category boundaries when continuous variables were categorized<br><br>**We did not categorize continuous variables.**

		(*c*) If relevant, consider translating estimates of relative risk into absolute risk for a meaningful time period<br><br>**Since we work in a probit regression setting, interpreting the effect of agriculture on the probability of malaria infection depends on other covariates in the model. Thus, we report our results in terms of risk differences. Specifically, we report the range or risk differences in children under 5 years of age, together with 95% uncertainty intervals, given a 15% increase in agricultural cover.**
Other analyses	17	Report other analyses done—eg analyses of subgroups and interactions, and sensitivity analyses<br><br>**We include the results and discussion from the other models considered in the supplementary appendix, and report the main results (i.e. those from the best-fitting model) in the main text.**
**Discussion**		
Key results	18	Summarise key results with reference to study objectives<br><br>**We provide a broad summary that our findings suggest that increasing agricultural coverage may lead to increases in malaria transmission, and that the mechanism may be through increased indoor biting among *An. gambiae* s.l. mosquitoes.**
Limitations	19	Discuss limitations of the study, taking into account sources of potential bias or imprecision. Discuss both direction and magnitude of any potential bias<br><br>**We discuss potential bias in our discussion. Specifically, we discuss that we treated temperature and precipitation as confounders, but that they may also mediate risk, with complex roles in transmission. We provide citations to work in this area.**<br><br>**Additionally, we note that it is likely impossible to draw a representative sample of a vector population over large land, and note this as a limitation,**

		**even though the vector data used in this study was sampled from different ecological zones.**
Interpretation	20	Give a cautious overall interpretation of results considering objectives, limitations, multiplicity of analyses, results from similar studies, and other relevant evidence<br><br>**We work to insure a cautious interpretation by using cautious language (i.e. the words "suggest" and "may"), e.g.:**<br><br>**-" Our data suggest increased malaria risk with increasing agriculture."**<br><br>**-"Results from entomological analyses suggest that increases in agriculture are associated with increased probability of indoor biting among *An. gambiae* s.l. mosquitoes"**<br><br>**-" Given the high abundance of *An. gambiae* s.l., these results suggest that agriculture-malaria relationship may be mediated through effects on indoor biting among *An. gambiae* s.l."**
Generalisability	21	Discuss the generalisability (external validity) of the study results<br><br>**We note that one of the strengths of this study is that it relies on a population-based survey of children under 5 years of age, suggesting that the results are generalizable to the population of children under 5 years of age.**<br><br>**We further not that one strength of this work is that the mosquito population was sampled in different ecological zones, which facilitates generalizability (although, as noted, representatively sampling a vector population remains a challenge).**
**Other information**		
Funding	22	Give the source of funding and the role of the funders for the present study and, if applicable, for the original study on which the present article is based

We include the following statements in the manuscript:

The sponsors of the study had no role in study design, data collection, data analysis, data interpretation, or writing of the report. The corresponding author had full access to all the data in the study and had final responsibility for the decision to submit for publication. Parental consent for children's participation in the 2013-2014 Demographic and Health Surveys (DHS) was obtained by the DHS Program. The 2013-2014 DRC DHS was reviewed and approved by the Institutional Review Board (IRB) at ICF International—the implementing agency of the DHS—and the University of Kinshasa IRB (Comité d'Ethique de l'Ecole de Santé Publique de l'Université de Kinshasa). This study was also approved by the IRB at the University of North Carolina at Chapel Hill.

The authors acknowledge support from the National Institutes of Health (grant 5R01AI107949 to Steven R. Meshnick), the National Science Foundation (grant BCS-1339949 to Michael Emch), the Gates Foundation (grant OPP1161913 to Brian J. Reich). Mark Janko received support from the Royster Society of Fellows at UNC-CH. Mark Janko and Marc Peterson were supported by the Population Research Infrastructure Program awarded to the Carolina Population Center (P2C HD050924) by the *Eunice Kennedy Shriver* National Institute of Child Health and Development. Seth Irish is funded by the US President's Malaria Initiative.

*Give information separately for exposed and unexposed groups.

Note: An Explanation and Elaboration article discusses each checklist item and gives methodological background and published examples of transparent reporting. The STROBE checklist is best used in conjunction with this article (freely available on the Web sites of PLoS Medicine at http://www.plosmedicine.org/, Annals of Internal Medicine at http://www.annals.org/, and Epidemiology at http://www.epidem.com/). Information on the STROBE Initiative is available at www.strobe-statement.org.

Model Specifications for Models on Probability of Malaria Infection:

Our outcome of interest is each individual's PCR-diagnosed malaria status, a binary indicator taking the value 1 if an individual is infected with malaria, and 0 otherwise. Typically, binary data are handled using logistic regression. However, spatial models for point-referenced data become computationally intensive very quickly as the number of spatial locations increases. This computational burden is further increased due to the lack of conjugacy between the prior distributions for model parameters and the data likelihood in logistic regression. As such, we adopt a probit specification in which we introduce latent variables that are assumed to follow a normal distribution with unit variance. Such a specification also has a scientific rationale. For example, we can think of these latent variables as a propensity to become infected with malaria, with values above 0 indicating increased propensity to become infected with malaria, and vice versa. To see this connection, observe that we can represent the probability of malaria infection, given covariates, as coming from a linear model. For example, let y_i^* be the binary indicator for whether or not individual i (i in $1 \dots n$) has malaria. Then the probability of malaria infection is given by:

$$\begin{aligned} \Pr(y_i^* = 1|X) &= \Pr(x_i^T\beta + \epsilon_i > 0) \\ &= \Pr(x_i^T\beta > -\epsilon_i) \\ &= \Pr(\epsilon_i < x_i^T\beta) \\ &= \Phi(x_i^T\beta) \end{aligned}$$

Where $\Phi(\cdot)$ is the CDF of a standard normal distribution.

Analysis of DHS data consisted of fitting three hierarchical probit regression models, differing only in the correlation structures specified for the random effects. Below, we outline the MCMC procedure for drawing posterior samples from the full conditional distributions for all model parameters for each model. We begin with the hierarchical probit model in which the random effects are assumed to be independent across space, and we then introduce spatial correlation in these random effects, beginning with the intercept, and then extending this to model a spatially-varying slope as well via a separable model.

The basic setup for all these models is as follows. Let:

$$Y = X\beta + Z\theta + \epsilon$$

Where Y is an $n \times 1$ vector latent normal responses, X is an n$\times$ p row vector of covariates (including an intercept) for individual, β is a $p \times 1$ column vector regression coefficients linking the covariates to the response, Z is an $n \times q$ random effects design matrix, where q is the number of DHS clusters in the dataset, 331 in this analysis. θ is a $q \times 1$ random intercept that varies across DHS clusters. Finally, ϵ is a white noise error assumed to follow a standard normal distribution.

The hierarchical model can be written as follows:

$$Y|\beta, \theta, \sigma^2 \sim N(X\beta + Z\theta, I_n)$$

$$\beta|\sigma_\beta^2 \sim N(0, \sigma_\beta^2 I_p)$$

$$\theta|\sigma^2 \sim N\left(0, \sigma^2 I_q\right)$$

$$\sigma^2 \sim IG(a, b)$$

<u>MCMC procedure for multilevel probit with independently varying intercept:</u>

for j in 1:n.posterior.samples{

<u>Step 1:</u> Draw from full conditional distribution of latent normal random variable Y, as follows:

Let y_i^* denote the binary indicator observed, taking the value 1 if the respondent has malaria, and 0 otherwise. Updating the latent variable y_i proceeds from sampling from a truncated normal distribution:

$$f(y_i|rest) \sim \begin{cases} N(x_i^T\beta + z_i^T\theta, 1, upper = 0),\ if\ y_i{}^* = 0 \\ N(x_i^T\beta + z_i^T\theta, 1, lower = 0), if\ y_i{}^* = 1 \end{cases}$$

Where $upper = 0$ indicates sampling from a truncated standard normal distribution truncated above by 0, while $lower = 0$ indicates sampling from a standard normal truncated below by 0.

<u>Step 2:</u> Draw from full conditional distribution of β:

Define $\gamma = Y - Z\theta$

$$\beta|rest \sim N\left(\left((X^TX + \frac{I_p}{\sigma_\beta^2})^{-1}\right)X^T\gamma, \left(X^TX + \frac{I_p}{\sigma_\beta^2}\right)^{-1}\right)$$

<u>Step 3:</u> Draw from full conditional distribution of θ:

Define $\mu = Y - X\beta$

$$\theta|rest \sim N\left(\left(Z^TZ + \frac{I_q}{\sigma^2}\right)^{-1} Z^T\mu, \left(Z^TZ + \frac{I_q}{\sigma^2}\right)^{-1}\right)$$

<u>Step 4:</u> Draw from full conditional distribution of σ^2:

$$\sigma^2|rest \sim IG(a^*, b^*)$$

Where $a^* = a + \frac{q}{2}$ and $b^* = \frac{\theta^T\theta}{2} + b$.

}

<u>MCMC procedure for hierarchical spatial probit with spatially varying intercept:</u>

The spatial model has the same general form as the probit specification above, but with additional parameters introduced into incorporate spatial structure. The hierarchical model thus has the following form:

$$Y|\beta, \theta, \sigma^2 \sim N(X\beta + Z\theta, I_n)$$

$$\beta|\sigma_\beta^2 \sim N(0, \sigma_\beta^2 I_p)$$

$$\theta|\sigma^2, \phi \sim N(0, \sigma^2\Sigma(\phi))$$

$$\sigma^2 \sim IG(a, b)$$

$$\phi \sim U(\phi_a, \phi_b)$$

Where everything is as before, except the variance for the random effects θ, where we introduce spatial structure through $\Sigma(\phi)$, which is a $q \times q$ matrix of pairwise distances between DHS clusters whose correlation decays according to an exponential correlation function with parameter ϕ. The prior for ϕ is chosen such that unmeasured confounding is spatially correlated from between 100 meters and 225 kilometers, roughly 10% of the breadth of DRC. Samples from the posterior distributions for all model parameters can be obtained by using the following MCMC steps:

for 1 in j:n.posterior.samples{

Step 1: Draw from full conditional distribution of latent normal random variable Y. Same as before.

Step 2: Draw from full conditional distribution of latent normal random variable β. Same as before.

Step 3: Sample from full conditional distribution of θ

Define $\mu = Y - X\beta$

$$\theta|rest \sim N((Z^TZ + \sigma^2\Sigma(\phi)^{-1})^{-1}Z^T\mu, (Z^TZ + \sigma^2\Sigma(\phi)^{-1})^{-1})$$

Step 4: Sample from full conditional distribution of σ^2

$$\sigma^2|rest \sim IG(a^*, b^*)$$

Where $a^* = a + \frac{q}{2}$ and $b^* = \frac{\theta^T\Sigma(\phi)^{-1}\theta}{2} + b$.

Step 5: Sample from full conditional distribution of ϕ:

First transform ϕ to have support on the real line using:

$$\phi^* = \log((\phi - \phi_a)/(\phi_b - \phi))$$

Then draw a proposal ϕ_p^* from:

$$N(\phi^*, v(\phi^*)),$$

where $v(\phi^*)$ is a tuning variance. Then, back transform to obtain proposal draw, ϕ_p, using:

$$\phi_p = (\phi_b \exp(\phi_p^*) + \phi_a)/(1 + \exp(\phi_p^*))$$

Calculate log acceptance ratio:

$$LAR = \frac{1}{2}(\log(\det(\Sigma(\phi)) - \log(\det(\Sigma(\phi))) +$$

$$\frac{1}{2\sigma^2}\theta^T\left(\Sigma(\phi)^{-1} - \Sigma(\phi_p)^{-1}\right)\theta +$$

$$\phi_p^* - \phi^* +$$

$$2\log((1 + \exp(\phi^*)/(1 + \exp(\phi_p^*))$$

Update ϕ according to the following:

$$\text{if } (\log(\text{U}(0,1) < \text{LAR})$$

$$\phi = \phi_p,$$

$$\text{else } \phi = \phi$$

}

MCMC procedure for multilevel spatial probit with spatially varying intercept and slope:

As with the model for the spatial intercept, the model for the spatial intercept and slope process differs only in how the random effects are specified. The hierarchical model can be written as follows:

$$Y|\beta, \theta, \sigma^2 \sim N(X\beta + Z\theta, I_n)$$

$$\beta|\sigma_\beta^2 \sim N(0, \sigma_\beta^2 I_p)$$

$$\theta|H, \phi \sim N(0, \Sigma(\phi) \otimes H)$$

$$H \sim IW(d+1, I_d)$$

Where instead of a single spatial variance parameter, we represent the spatial variance-covariance matrix for the intercept and slope processes using the 2×2 matrix H (i.e. d=2 in the above specification). Note here too that the random effects design matrix Z is now $n \times 2q$, with the additional q columns containing the agricultural exposure around each DHS cluster. We specify an Inverse Wishart distribution with 3 degrees of freedom and a 2×2 identity scale matrix as the prior. Samples from the posterior distributions for all model parameters can be obtained by using the following MCMC steps:

for j in 1:n.posterior.samples{

Step 1: Draw from full conditional distribution of latent normal random variable Y. Same as before.

Step 2: Draw from full conditional distribution of latent normal random variable β. Same as before.

Step 3: Draw from full conditional distribution of θ:

$$f(\theta|rest) \sim N((Z^TZ + \Sigma(\phi)^{-1} \otimes H^{-1})^{-1} Z^T \mu, (Z^TZ + \Sigma(\phi)^{-1} \otimes H^{-1})^{-1})$$

Step 4: Draw from full conditional distribution of ϕ:

First transform ϕ to have support on the real line using:

$$\phi^* = \log((\phi - \phi_a)/(\phi_b - \phi))$$

Then draw a proposal ϕ_p^* from:

$$N(\phi^*, v(\phi^*)),$$

where $v(\phi^*)$ is a tuning variance. Then, back transform to obtain proposal draw, ϕ_p, using:

$$\phi_p = (\phi_b \exp(\phi_p^*) + \phi_a)/(1 + \exp(\phi_p^*))$$

Calculate log acceptance ratio:

$$LAR = \frac{1}{2}(\log(\det(\varSigma(\phi) \otimes H) - \log(\det(\varSigma(\phi) \otimes H)) +$$

$$\frac{1}{2\sigma^2}\theta^T \left(\Sigma(\phi)^{-1} \otimes H^{-1} - \Sigma(\phi_p)^{-1} \otimes H^{-1}\right)\theta +$$

$$\phi_p^* - \phi^* +$$

$$2\log((1 + \exp(\phi^*)/(1 + \exp(\phi_p^*))$$

Update ϕ according to the following:

$$\text{if } (\log(\mathrm{U}(0,1) < \mathrm{LAR})$$

$$\phi = \phi_p,$$

$$\text{else } \phi = \phi$$

Step 5: Draw from full conditional distribution of H:

$$H|rest \sim IW(q+3, \theta^T\Sigma(\phi)^{-1}\theta + I_2)$$

}

All models were run for 120,000 iterations, with the first 20,000 discarded as burn-in and the Markov chain thinned such that inference about model parameters is based on 10,000 posterior samples. Model convergence was assessed by inspecting traceplots of model parameters, and final inferences are based on the best fitting model.

Model fitting results

Fit statistic	*Random Intercept*	*Spatial Random Intercept*	*Spatial Random Intercept and slope*
Brier score	0.160	0.161	0.159
ROC curve	0.839	0.836	0.838
DIC	4687	4695	4715

Supplementary Table 1. Fit statistics for hierarchical probit regression models on agriculture and malaria risk

Spatial models were initially compared by randomly withholding a third of the spatial locations and predicting those data out-of-sample. Performance was identical across both models, as can be seen above, and all models were re-fit to the full data, with final inferences presented in the manuscript being based off of the model incorporating a random intercept, as it had the lowest DIC.

While the non-spatial model exhibited the best fit to the data, we show results for the spatial processes from both models here, as these can be suggestive of potential areas of future concern. Supplementary Figure 1 below shows the spatial intercept surface, together with corresponding uncertainty.

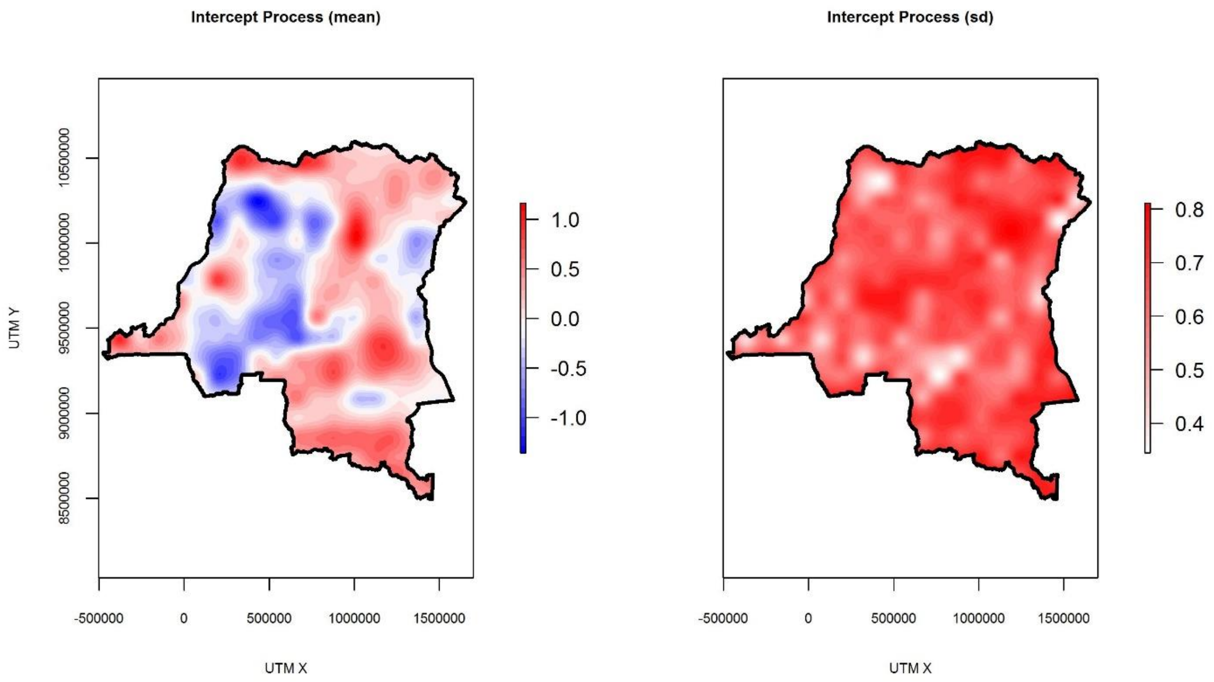

Supplementary Figure 1: Maps of the mean spatial random intercept (left) and its corresponding uncertainty (right), represented as standard deviation from the mean.

Considerable variability in the spatial random intercept process persists after accounting for other risk factors, with areas of the DRC exhibiting both strong increased and decreased risk of infection, particularly in northern regions. Notably, however, these estimates are accompanied by considerable imprecision, preventing definitive conclusions about areas of increased or decreased residual risk.

Supplementary Figure 2 below shows the spatial intercept and slope surfaces for the model incorporating both a spatially varying intercept and a spatially varying slope for the effect of agriculture on malaria risk.

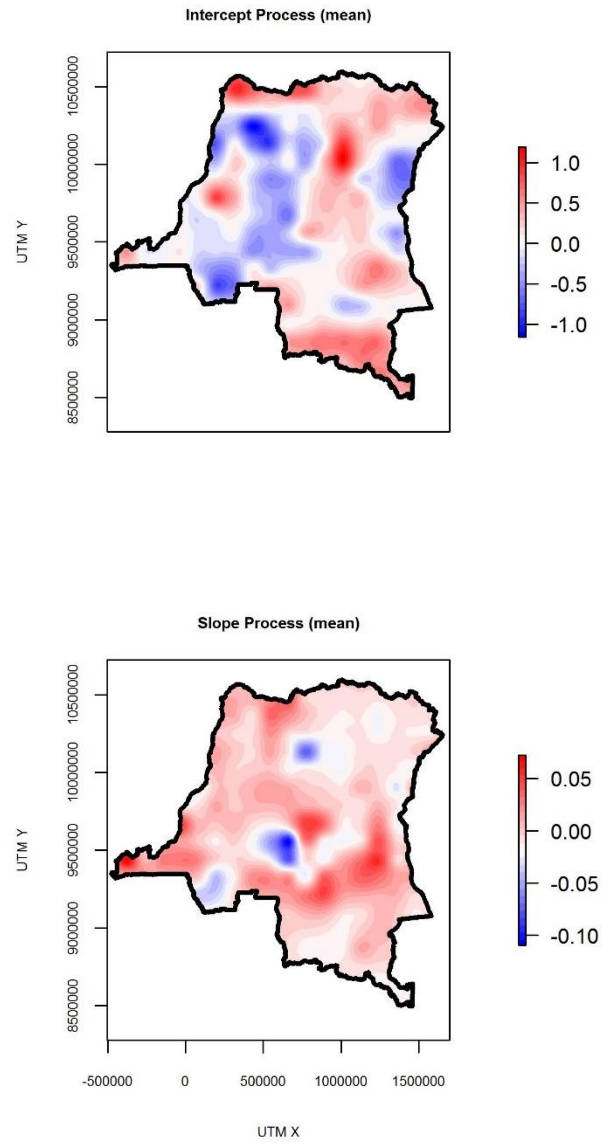

Supplementary Figure 2: Maps of the mean spatial random intercept (top) and slope processes (bottom), together with their corresponding uncertainty (top and bottom right, respectively), represented as standard deviation from the mean.

Incorporating the spatial random slope leads to slight attenuation in the intercept process, although the spatial pattern broadly remains. Further, there is slight evidence of possible attenuation of the effect of agriculture in two places in

DRC, one in the central-northern region, which is largely forest, and the other in central DRC in what is largely Savannah. This latter area also shows pockets of increased risk. In both cases, however, inferences on the intercept and slope processes are accompanied by considerable imprecision
